# Impact of Na Doping on the Carrier Transport Path in Polycrystalline Flexible Cu_2_ZnSn(S,Se)_4_ Solar Cells

**DOI:** 10.1002/advs.201903085

**Published:** 2020-09-27

**Authors:** Woo‐Lim Jeong, Kyung‐Pil Kim, Juran Kim, Ha Kyung Park, Jung‐Hong Min, Je‐Sung Lee, Seung‐Hyun Mun, Sung‐Tae Kim, Jae‐Hyung Jang, William Jo, Dong‐Seon Lee

**Affiliations:** ^1^ School of Electrical Engineering and Computer Science Gwangju Institute of Science and Technology Gwangju 61005 Republic of Korea; ^2^ Research Institute for Solar and Sustainable Energies Gwangju 61005 Republic of Korea; ^3^ Department of Physics and New and Renewable Energy Research Center Ewha Womans University Seoul 03760 Republic of Korea

**Keywords:** CZTSSe, thin‐film solar cells, flexible electronics, doping, carrier transport

## Abstract

It is well‐known that the alkali doping of polycrystalline Cu_2_ZnSn(S,Se)_4_ (CZTSSe) and Cu(In,Ga)(Se,S)_2_ has a beneficial influence on the device performance and there are various hypotheses about the principles of performance improvement. This work clearly explains the effect of Na doping on the fill factor (FF) rather than on all of the solar cell parameters (open‐circuit voltage, FF, and sometimes short circuit current) for overall performance improvement. When doping is optimized, the fabricated device shows sufficient built‐in potential and selects a better carrier transport path by the high potential difference between the intragrains and the grain boundaries. On the other hand, when doping is excessive, the device shows low contact potential difference and FF and selects a worse carrier transport path even though the built‐in potential becomes stronger. The fabricated CZTSSe solar cell on a flexible metal foil optimized with a 25 nm thick NaF doping layer achieves an FF of 62.63%, thereby clearly showing the enhancing effect of Na doping.

## Graphical abstract

The effects of Na doping on polycrystalline flexible Cu_2_ZnSn(S,Se)_4_ (CZTSSe) solar cells are analyzed. When doping is excessive, the device shows low contact potential difference and fill factor and selects a worse carrier transport path even though the built‐in potential becomes stronger. On the other hand, the optimized CZTSSe solar cell clearly shows the enhancing effect of Na doping.

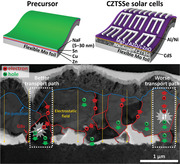

## 1 Introduction

Polycrystalline Cu(In,Ga)(Se,S)_2_ (CIGS) solar cells have advantages such as high thermal stability and being both lightweight and flexible. Many studies are being conducted by various companies and laboratories to surpass the performance of Si solar cells. Especially, most of the leading contenders with power conversion efficiencies (PCEs) of more than 20% are using alkali postdeposition and alkali‐containing film to improve performance.^[^ ^1^, ^2^, ^3^, ^4^ ^]^ Many studies on Cu_2_ZnSn(S,Se)_4_ (CZTSSe) solar cells are also being conducted by applying alkali doping, which has the advantage of using earth‐abundant and low‐cost elements compared to CIGS solar cells.^[^ ^5^, ^6^, ^7^, ^8^, ^9^, ^10^, ^11^, ^12^, ^13^, ^14^ ^]^ In addition, alkali doping is essential when flexible substrates are employed in place of the more commonly used rigid soda‐lime glass (SLG) due to Na and K diffusion effects.^[^ ^15^, ^16^, ^17^, ^18^, ^19^, ^20^, ^21^, ^22^, ^23^, ^24^, ^25^, ^26^, ^27^, ^28^, ^29^ ^]^ López‐Marino et al.^[^ ^15^ ^]^ compared alkali doping methods such as MoNa back contact, NaF and KF layers with a 6.1% PCE of flexible Cu_2_ZnSnSe_4_ solar cells. They achieved the highest efficiency when using MoNa back contact, but the contents of alkali elements of each doping method were not strictly controlled. Sun et al.^[^ ^18^ ^]^ achieved 6.2% PCE for quaternary Cu_2_ZnSnS_4_ solar cells on the flexible stainless steel substrate and reported an improvement in adhesion of MoNa back contact layer. If the MoNa layer directly touches the substrate or the absorbing layer, it leads to bad adhesion or interface recombination, so they solved it by adding the Mo layer on both sides of the MoNa layer. Interestingly, Jo et al.^[^ ^16^ ^]^ obtained 8.0% PCE for flexible CZTSSe solar cells due to high short circuit current (*J*_SC_) and open‐circuit voltage (*V*_OC_) without alkali doping, but a fill factor (FF) was low. Yang et al.^[^ ^20^ ^]^ reported 10.34% PCE for flexible CZTSSe solar cells, which is considered the record efficiency, and its enlarging methods using multilayered precursor structure. They inserted a 10 nm thick NaF doping layer between the Mo foil and the precursor layer without precise Na content control. Generally, it is known that alkali elements avoid becoming recombination centers, form benign secondary phases, help minority carrier transportation through grain‐boundary (GB) passivation, and prevent the formation of deep‐level defects.^[^ ^30^, ^31^, ^32^ ^]^ Although mainstream principles have been elucidated, there is still much debate on how alkali doping leads to performance improvement.^[^ ^33^ ^]^ We fabricated a CZTSSe solar cell with an FF of over 60% on a flexible metal substrate and used it to analyze the effects of Na doping. From the results, we postulate that the carrier transport path depends on the amount of Na dopant, which affects the electrical resistance and FF of the polycrystalline CZTSSe solar cells.

## 2 Results and Discussion

**Figure 1:**
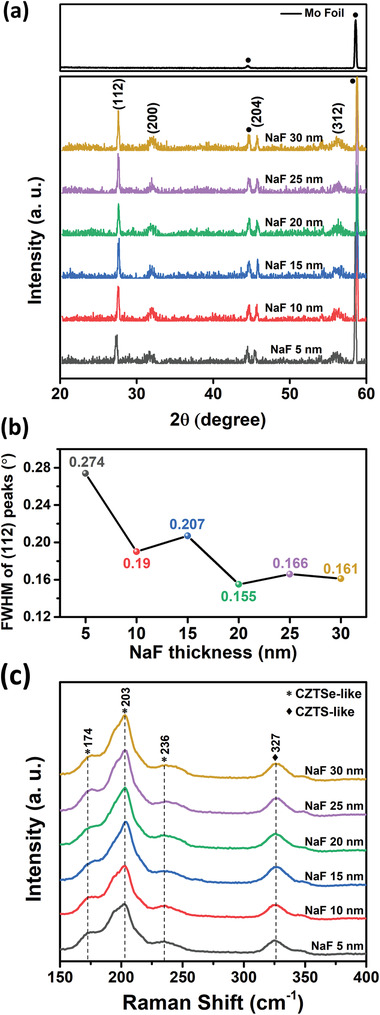
a) X‐ray diffraction (XRD) patterns, b) full‐width at half‐maximum (FWHM) of the (112) peaks from the XRD data, and c) Raman spectra of the CZTSSe absorbing layers fabricated with NaF layers of varied thicknesses on flexible Mo foils.

X‐ray diffraction (XRD) analysis was conducted to examine the crystallinity of the CZTSSe absorbing layers according to the varied Na doping conditions, the results of which are shown in **Figure** **1**a. The X‐rays used in the XRD analysis had a high penetration depth, and so the CZTSSe absorbing layer and Mo foil substrate were both detected. All samples attained CZTSSe peaks indicating the (112), (200), (204), and (312) planes. full‐width at half‐maximum (FWHM) values of the predominant (112) peak for each sample are exhibited in Figure 1b; the CZTSSe absorbing layer showed better crystallinity as the NaF doping layer thickness increased, although the FWHM value became nearly saturated at an NaF layer thickness of 20 nm or higher. Na atoms are known to help form large grains in CZTSSe absorbing layers,^[^ ^8^ ^]^ which was also indicated by the results of a surface scanning electron microscopy (SEM) analysis (Figure S1, Supporting Information). To analyze the presence of secondary phases in the CZTSSe layer surface, Raman measurements were conducted using a 514 nm wavelength laser with a penetration depth of around 100 nm. The size of the CZTSe peak (203 cm^−1^) was larger than the CZTS peak (327 cm^−1^) because the Se content was larger than the S content. The 5 and 10 nm thick NaF layer samples, in which Na doping was insufficient, showed low intensity at 203 cm^−1^ along with broadening due to low crystallization and atomic defects. In addition, the results of an energy‐dispersive X‐ray spectroscopy (EDX) analysis on the CZTSSe absorber samples show optimized Zn‐rich and Cu‐poor stoichiometry (Table S1, Supporting Information), which is in accordance with our previous research.^[^ ^34^ ^]^

**Figure 2:**
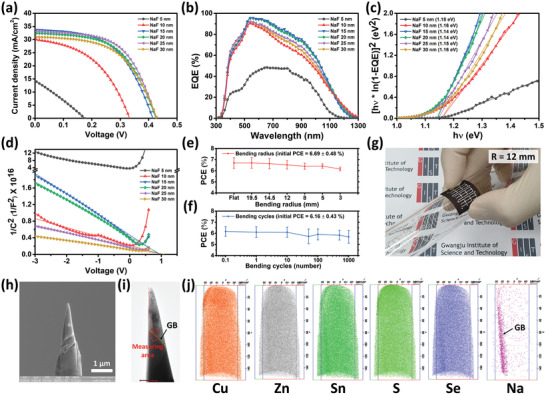
a) Current density–voltage (*J–V*), b) external quantum efficiency (EQE), c) the bandgap obtained from the EQE data, and d) capacitance–voltage (*C*–*V*) measurements of the CZTSSe solar cells on flexible Mo foil fabricated using different NaF layer thicknesses. Bending test results for e) a bending radius from flat to 3 mm and f) bending cycles using a bending radius of 12 mm. Error bars indicate standard error of the mean (*n* = 9). g) a digital camera image of a flexible CZTSSe solar cell. h) SEM and i) TEM images of the CZTSSe solar cell sample with a 30 nm thick NaF layer for atom probe tomography (APT). j) 3D atomic maps of various elements.

The flexible CZTSSe solar cells were completed using CZTSSe absorbing layers with different amounts of Na dopant; the analysis of their electrical characteristics are exhibited in **Figure** **2**a and reported in Table S2 in the Supporting Information. CZTSSe solar cells with 5 and 10 nm thick NaF layers (insufficient Na doping) showed high *E*_g_ */q − V*_OC_ (*V*_OC_ deficit) and low FF values. On the other hand, CZTSSe solar cells with 15–30 nm thick NaF layers (sufficient Na doping) showed high PCE values of over 7% and only a tiny difference in each solar cell parameter. Among them, the one with a 15 nm thick NaF layer had the highest *J*_SC_ value which then slightly decreased as the Na doping increased. Moreover, the *V*_OC_ was maintained at 0.427 V when the amount of Na dopant was sufficient. The CZTSSe solar cell with a 25 nm thick NaF layer showed the highest FF value which we think is due to the difference in the transport path of the electron–hole pair separated by the CZTSSe absorbing layer to both electrodes. The best‐performing cell was certified by an accredited laboratory (Korea Institute of Energy Research, KIER) who reported an FF of 62.63% and a PCE of 8.66% (Figure S2, Supporting Information). For verification of the statistical distribution, PCE histograms of the nine solar cells are shown in Figure S3 in the Supporting Information. The results of an external quantum efficiency (EQE) analysis and estimated bandgap energy graph can be found in Figure 2b,c, respectively. The flexible CZTSSe solar cells showed bandgap energy of 1.14–1.18 eV. In general, the dominant modes of the carrier separation of crystalline Si and thin‐film solar cells are diffusion and drift, respectively. Therefore, the *C*–*V* measurement at the pn‐junction of the CZTSSe thin‐film solar cells is quite useful for analyzing the electrostatic field and drift force (Figure 2d and **Table** **1**). The relationship between the doping density of the acceptor (*N*_a_) and the built‐in potential (*V*_bi_) at the pn‐junction can be derived as follows^[^ ^35^, ^36^ ^]^

(1) $$\begin{array}{c} N_{a}=\frac{2}{q\varepsilon_{0}\varepsilon_{s}A^{2}\left[\frac{d}{dV}\left(\frac{1}{C^{2}}\right)\right]} \end{array}$$

(2) $$\begin{array}{c} \frac{1}{C^{2}}=\frac{2}{qN_{a}\varepsilon_{0}\varepsilon_{s}A^{2}}\;\left(V_{\mathrm{bi}}-V\right) \end{array}$$

where *q* is the electron charge (1.60219 × 10^−19^ C), *ε*_0_ is the permittivity of free space (8.85 × 10^−14^ F cm^−1^), *ε*_s_ is the dielectric constant (that of CZTSSe with bandgap energy of 1.1 eV is 8^[^ ^37^ ^]^), *A* is the area of the cell (cm^2^), *C* is the measured capacitance, and *V* is the applied DC voltage. The increase in *V*_bi_ was according to the following equation related to the pn‐junction between the p‐type and n‐type semiconductors

(3) $$\begin{array}{c} V_{\mathrm{bi}}=\frac{\mathrm{kT}}{q}\;\ln\left(\frac{N_{a}N_{d}}{n_{i}^{2}}\right) \end{array}$$

where *k* is Boltzmann's constant, *T* is the temperature, *N*_a_ is the doping density of the acceptor, *N*_d_ is the doping density of the donor, and *n*_i_ is the intrinsic concentration of electrons. In the case of the CZTSSe solar cells, the Na atoms act as an acceptor. Therefore, as the amount of Na atoms increases, *N*_a_ and *V*_bi_ increase. Except for the solar cells with 5 and 10 nm thick NaF layers which have nonlinear characteristics, all of the others naturally showed increased *N*_a_ and *V*_bi_ as the NaF layer thickness increased. This increase in *V*_bi_ is known to lead to better carrier separation and improved PCE due to a strong electrostatic field, but the solar cell with a 30 nm thick NaF layer showed lower PCE than the one with a 25 nm thick NaF layer. To verify the bending stability of the flexible CZTSSe solar cells fabricated on the Mo foil, bending tests for radius and durability were conducted using samples with an average of over 6% initial PCE, results of which are shown in Figure 2e–g. When samples were bent to a radius of 3 mm and then allowed to return, they maintained 91.9% performance (PCE = 6.15% ± 0.17%) compared to their initial performance (PCE = 6.69% ± 0.48%). In addition, for a bending radius of 12 mm and for 1000 bending cycles, the samples maintained 92.2% performance (PCE = 5.68% ± 0.57%) compared to the untested samples (PCE = 6.16% ± 0.43%). In addition, atom probe tomography (APT) conducted on a 30 nm sample provides clear evidence for the distribution of Na atoms near GBs (Figure 2h–j).

**Table 1:** Doping density of acceptor and built‐in potential of the CZTSSe solar cells with varied NaF layer thicknesses obtained from the *C*–*V* measurements with an analysis near the pn‐junction

NaF thickness [nm]	5	10	15	20	25	30
*N*_a_ [atom cm^−3^]	1.93 × 10^16^	2.52 × 10^17^	1.10 × 10^17^	1.24 × 10^17^	3.16 × 10^17^	5.03 × 10^17^
*V*_bi_ [V]	1.06	0.99	0.92	0.97	1.02	1.1

**Figure 3:**
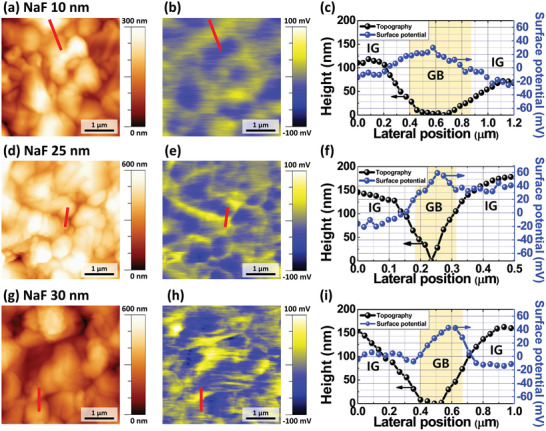
Surface topography, potential characteristics, and line profiles of the CZTSSe absorbing layers with a–c) 10 nm, d–f) 25 nm, or g–i) 30 nm NaF layer thicknesses determined by Kelvin probe force microscopy (KPFM) measurements.

From the results of the previous analyses, it was found that the sample with a 30 nm thick NaF layer showed lower PCE than the one with a 25 nm thick NaF layer even though it had a higher *V*_bi_. Thus, Kelvin probe force microscopy (KPFM) measurements were conducted for the intragrain regions (IGs) and GBs of the CZTSSe absorbing layer, as shown in **Figure** **3**. Thereafter, the samples with NaF layers of 10, 25, and 30 nm thicknesses were analyzed for insufficient, optimum, and excessive Na doping, respectively. All samples showed high surface potential in the vicinity of the GBs and low surface potential at the IGs (Figure 3c,f,i), but the level of the potential difference varied depending on the amount of Na dopant. The sample with a 10 nm thick NaF layer showed weak band bending whereas those with 25 and 30 nm thick NaF layers showed strong band bending between the IGs and GBs in the CZTSSe absorbing layer.

**Figure 4:**
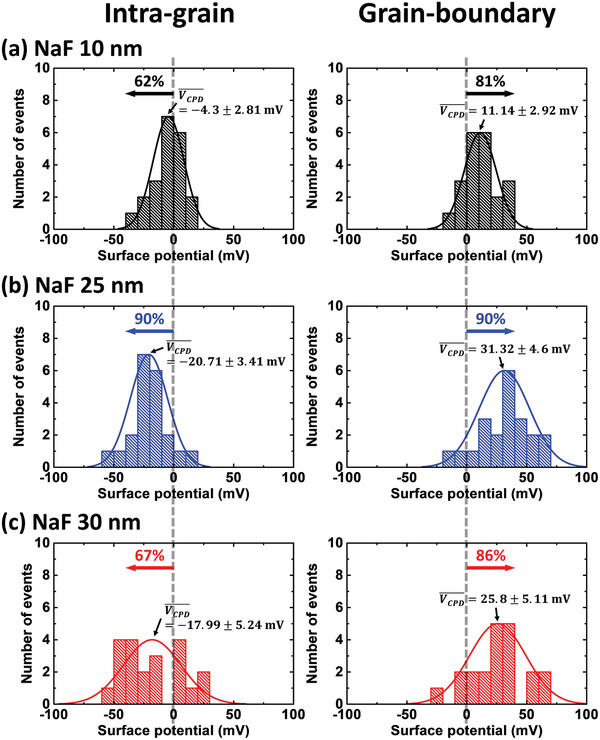
Histograms of the intra‐grain (IG) and grain‐boundary (GB) surface potential distributions of the CZTSSe absorbing layers with a) 10 nm, b) 25 nm, or c) 30 nm NaF layer thicknesses obtained from the line profile data of the KPFM measurements (*n* = 21). For statistical analysis, one‐way analysis of variance (ANOVA) was used for GB and IG, respectively. Data are presented as mean ± standard error of the mean from three samples.

For more precise analysis, KPFM measurements were conducted with the focus on the IGs and GBs; surface potential distribution graphs and mean values of the contact potential difference (*V*_CPD_) are shown in **Figure** **4**. When Na doping was insufficient (a 10 nm thick NaF layer), the smallest *V*_CPD_ of −4.3 ± 2.81 and 11.14 ± 2.92 mV were measured at the IGs and GBs, respectively. On the other hand, these values were the largest (−20.71 ± 3.41 and 31.32 ± 4.6 mV, respectively) when Na doping was optimum (a 25 nm thick NaF layer) but reduced to −17.99 ± 5.24 and 25.8 ± 5.11 mV, respectively, for excessive Na doping (a 30 nm thick NaF layer). The probable reason for the decrease in *V*_CPD_ of the NaF 30 nm sample is that the Na dopant led to the formation of negatively charged defects (Na_Cu_ and Na_Zn_) near the GBs rather than relatively positive ones (Na_i_, Na_S_, Na_Se_, and Na_Sn_).^[^ ^38^, ^39^, ^40^ ^]^ Some defects in CZTSSe solar cells are generally known to have detrimental effects such as band tailing and *V*_OC_ loss. However, Na‐related defects can prevent the formation of deep‐level defects and contribute to performance improvements by helping carrier transport. These Na‐related defects are typically Na_Cu_^0^, Na_Zn_^−^, Na_Sn_^3−^, Na_i_^+^, and Na_Se_^+^.^[^ ^39^ ^]^ Na_Cu_^0^ and Na_Zn_^−^ are negatively charged while Na_i_^+^ and Na_Se_^+^ are naturally positively charged. Na_Sn_^3−^ is an exceptional charge localized state where the electrons are strongly bound to the defect and difficult to ionize. Therefore, they are divided into positively charged (Na_i_, Na_S_, Na_Se_, and Na_Sn_) and negatively charged (Na_Cu_ and Na_Zn_) defects. Returning to our results, we identified the surface potentials according to the Na content through KPFM. When Na doping was insufficient, the smallest *V*_CPD_ was measured. Here, both positively and negatively charged defects would have been suppressed (=low *V*_CPD_). However, the formation of positively charged defects was maximized and that of negatively charged ones was minimized when Na doping was optimized (=highest *V*_CPD_). As a result of the statistical analysis, NaF 10 nm versus NaF 25 nm (*p* < 0.05) and NaF 10 nm versus NaF 30 nm (*p* < 0.05) showed significant differences but NaF 25 nm versus NaF 30 nm (*p* = 0.63) did not at the GBs due to the fine control of the NaF layers. The results of the statistical analysis at the IGs showed a similar tendency (*p* < 0.05, *p* < 0.05, and *p* = 0.87). If the doping became excessive, negatively charged defects also increased (=low *V*_CPD_). In addition, the results of a conductive atomic force microscopy (c‐AFM) analysis on the same three samples also show that the strongest local current was in the vicinity of the GB when Na doping was optimum (Figure S4, Supporting Information). Histograms of the KPFM analysis results for the remaining three samples are also shown in Figures S5 and S6 in the Supporting Information. To identify the elemental distributions and Na content in the three samples, EDX mapping for a transmission electron microscopy (TEM) analysis and secondary ion mass spectrometry (SIMS) were performed (Figure S7, Supporting Information). No noticeable secondary phases were detected in all three samples, although a Cd‐rich region formed near the GBs and voids. It seems that the Cd atoms replaced the Zn atoms near the GBs to form Cu_2_(Zn,Cd)Sn(S,Se)_4_ (Figure S8, Supporting Information) and they do not seem to have adversely affected the device performance.^[^ ^41^, ^42^ ^]^ In the SIMS analysis, the Na atoms were mainly distributed in the CZTSSe/Mo(S,Se) interface and were detected up to 5 × 10^3^ times in the 30 nm NaF‐thick sample (Figure S9, Supporting Information); More Na atoms were detected as the thickness of the NaF layer increased. Na atoms were mainly distributed near the GBs. In addition, cross‐sectional SEM images used to verify the thickness of the Mo(S,Se) layers in the flexible CZTSSe solar cells are shown in Figure S10 in the Supporting Information. A thick MoSSe layer is usually considered to be detrimental to device performance. However, some studies on high‐efficiency CZTSSe solar cells have obtained more than 10% PCE with quite thick MoSSe layers that did not lead to significant performance degradation.^[^ ^20^, ^43^ ^]^

**Figure 5:**
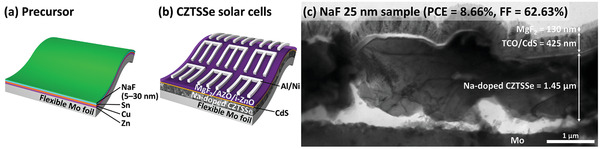
Schematic diagrams of a) the metallic precursor with the NaF doping layer and b) CZTSSe solar cells on the flexible Mo foil. c) A cross‐sectional TEM image of the best‐performing CZTSSe solar cell.

Based on the results of the analyses, we fabricated an optimized flexible CZTSSe solar cell using the metallic precursor and a 25 nm thick NaF doping layer which could attain a PCE of 8.66% and an FF of 62.63%. Schematic diagrams and a cross‐sectional TEM image of the best‐performing cell are shown in **Figure** **5**. The results of a comparison of other reported CZTSSe solar cell characteristics with the results of this work are summarized in **Table** **2**. Very recently, Yang et al.^[^ ^20^ ^]^ announced the most efficient flexible CZTSSe solar cell (PCE = 10.34%, *V*_OC_ = 0.513 V, *J*_SC_ = 35.23 mA cm^−2^, and FF = 57.2%) which was certified by the KIER. Note that our flexible CZTSSe solar cell was certified by the same institution and showed a higher FF even though the PCE was lower. Therefore, our results clearly show the effect of Na doping on the FF of solar cells.

**Table 2:** Comparison of the characteristics of some reported flexible CZTSSe solar cells and world record CZTSSe solar cells on a rigid SLG substrate

Institute (Country)	Material	Alkali‐doping method	Substrate	PCE [%]	FF [%]	Ref.
IREC (Spain)	CZTSe	MoNa	SS 430	6.1	56.8	^[^ ^15^ ^]^
UNSW (Australia)	CZTS	MoNa	SS 430	6.29	57.51	^[^ ^18^ ^]^
Fuzhou University (China)	CZTSSe	Annealing with SLG	Mo foil	6.78	57.71	^[^ ^19^ ^]^
Daegu Gyeongbuk Institute of Science and Technology (South Korea)	CZTSSe	NaF	Mo foil	10.34	57.22	^[^ ^20^ ^]^
GIST (South Korea)	CZTSSe	NaF	Mo foil	8.66	62.63	This work
IBM (USA)	CZTSSe	–	Rigid SLG	12.6	69.8	^[^ ^44^ ^]^

**Figure 6:**
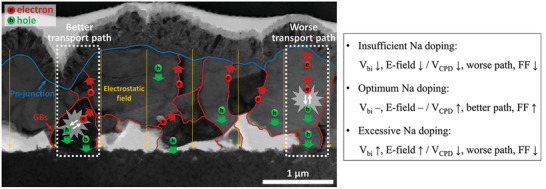
Schematic representation of the effect of Na doping on the electron–hole transport path illustrated on a cross‐sectional transmission electron microscopy (TEM) image of the CZTSSe solar cell sample with a 30 nm thick NaF layer.

In accordance with the results reported so far, a schematic representation of the effect of Na doping on the CZTSSe solar cells is shown in **Figure** **6**. The incident photons are absorbed by the polycrystalline CZTSSe layer and form electron–hole pairs. Among these carriers, the electrons move to the top electrode and the holes move to the bottom one under the influence of the electrostatic field to generate the electric current. At this stage, the electrons are expected to move near the GBs, while the holes are expected to move toward the IGs simultaneously due to the upward *V*_CPD_ bending for better carrier transport. In general, it is known that the defects near GBs are passivated by Na atoms as they form higher p‐type conductivity regions rather than recombination centers.^[^ ^14^, ^17^, ^45^ ^]^ We can confirm from the *C*–*V* measurement that the *V*_bi_ increases as the amount of Na dopant increases, so the strength of the electrostatic field also increases. According to this explanation, PCE is increased as the electrostatic field becomes stronger, but this was not the case in the actual measurement results. Although the interstitial Na dopant in GBs is known to weaken the bonds of deep‐level defects and have beneficial effects on a device,^[^ ^32^ ^]^ excessive Na dopant does not fulfill that role and instead decreases the *V*_CPD_. Moreover, excessive Na contents can cause an impurity in the buffer layer, resulting in recombination at interfaces.^[^ ^46^ ^]^ Thus, the carriers were collected most likely via a worse transport path with low p‐type conductivity, thereby reducing the PCE. In addition, the electrons move through the inside of the CZTSSe crystal instead of in the vicinity of the GBs and either carrier recombination occurs or the series resistance increases (Table S2, Supporting Information), thereby reducing the FF. On the other hand, when Na doping was optimized, the formation of positively charged defects (Na_i_, Na_S_, Na_Se_, and Na_Sn_) near the GBs was maximized and negatively charged ones (Na_Cu_ and Na_Zn_) was minimized. Therefore, *V*_CPD_ was maximized, which was confirmed from the KPFM measurements. High *V*_CPD_ helps the carriers to choose a highly conductive p‐type path and reduces the series resistance. It can also be interpreted that the carrier recombination is reduced because electrons and holes travel different paths.^[^ ^47^ ^]^ As a result, the FF and PCE of the sample with a 25 nm thick NaF layer were improved (as can be seen in the IV curves).

## 3 Conclusion

We demonstrated the effect of Na doping on polycrystalline CZTSSe solar cells through various measurements and analyzed the principle of carrier transport. First of all, the crystallinity of the CZTSSe absorbing layers according to Na dopant content was determined via an XRD analysis and was then confirmed by the FWHM of the (112) peaks becoming saturated when the thickness of the NaF layer was greater than 20 nm. From the *J–V* analysis, the flexible CZTSSe solar cell fabricated using a 25 nm thick NaF layer achieved a PCE of 8.66% and the highest FF of 62.63%. In addition, the *V*_bi_ at the pn‐junction (which is a major force affecting carrier separation) increased from 0.92 to 1.1 V within valid samples as the doping level increased. After 1000 bending cycles with a bending radius of 12 mm, our flexible CZTSSe solar cells maintained 92.2% of the initial PCE. In addition, KPFM measurement revealed that the sample with a 25 nm thick NaF layer attained a higher *V*_CPD_ (−20.71 and 31.32 mV) than that of the 30 nm thick NaF layer sample for IGs and GBs, respectively. Based on the results, we deduced that the carriers are collected through a better transport path with sufficient electrostatic field strength when the Na doping is optimized. On the other hand, when the Na doping is excessive, we surmised that the carriers are collected through a worse transport path due to the low *V*_CPD_, so the device obtained lower FF and PCE values despite the stronger electrostatic field strength. These results are from the analysis of polycrystalline CZTSSe solar cells formed on flexible Mo foil, but we expect a similar interpretation of the mechanism in polycrystalline CIGS solar cells, which are almost identical to the CZTSSe solar cell structure. We also believe that this work will broaden the understanding of these polycrystalline thin‐film solar cells and help improve the FF even further.

## 4 Experimental Section

##### Formation of the CZTSSe Absorbing Layers on Mo Foil

flexible Mo foils (99.95%, 0.1 mm thick; Kojundo Chemical Laboratory Co., Ltd., Japan) used as the back electrode and substrate were cleaned in acetone, methanol, and deionized water for 15 min each. After this, Zn, Cu, and Sn layers were sequentially deposited on the Mo foil: DC magnetron sputtering was used for the deposition of the Zn and Cu layers and radio frequency magnetron sputtering for the Sn layer. Each metal layer was deposited using 50 W (1.1 W cm^−2^) sputtering power in an argon atmosphere at a pressure of 0.4 Pa. Deposition times of the metallic layers were determined as the Zn‐rich and Cu‐poor EDX composition ratio of the absorbing layer. Very thin NaF doping layers from 5 to 30 nm thick were deposited on the metallic precursors using an electron beam evaporator. The precursors were sulfoselenized with 0.5 g of Se and 0.04 g of SeS_2_ powders in the graphite box using rapid thermal annealing (RTA). The RTA chamber was evacuated for 3 min using a rotary pump before the annealing process to remove any unwanted atoms and then filled with argon gas to atmospheric pressure. In the annealing process, the precursors were heated from room temperature to 300 °C in 1000 s and then maintained at 300 °C for 1500 s. Next, they were heated from 300 to 560 °C in 1000 s and then maintained at 560 °C for 1100 s.

##### Fabrication of the Buffer, Window Layer, Electrode, and Antireflection Coating

The CZTSSe absorbing layers on flexible Mo foil were immersed in 0.05 mol aqueous potassium cyanide solution for 5 min to remove the secondary phase and then rinsed with deionized water. Afterward, a 70 nm CdS buffer layer was deposited by chemical bath deposition at 80 °C. 50 nm intrinsic ZnO and 300 nm aluminum‐doped ZnO window layers were deposited on the samples using radio frequency magnetron sputtering at room temperature and 150 °C, respectively. 50 nm Ni and 500 nm Al electrodes were deposited through a shadow mask with an electron beam evaporator followed by deposition of a 130 nm thick MgF_2_ antireflection coating.

##### Characterization

The structural properties of the absorbing layers were analyzed using XRD (X'pert‐APD, Philips, USA) with Cu*K*_*α*_ radiation. Raman scattering measurement was performed with laser excitation wavelengths at 514 nm. SEM (S‐4700, Hitachi, Japan) and EDX were used for the surface analysis of the CZTSSe absorbing layers. The PCE and solar cell parameters for CZTSSe solar cells were measured with a class AAA solar simulator (Oriel Sol 3A, Newport, USA) under conditions of AM 1.5G, 100 mW cm^−2^, and 25 °C. Afterward, the best‐performing certified cell was measured with a different class AAA solar simulator (WXS‐155S‐L2, Wacom, Japan). The EQE and *C*–*V* measurements were performed with a QEX7 Solar Cell Spectral Response/Incident Photon‐to‐electron Conversion Efficiency/Quantum Efficiency Measurement System (PV Measurements, USA) and a precision LCR meter (4284A, Agilent Scientific Instruments, USA) at room temperature, respectively. Bending tests on the flexible CZTSSe solar cells were conducted manually using cylindrical objects. The APT measurements were performed using local electrode atom probes (LEAP 4000X HR, Cameca, USA). Commercial c‐AFM (n‐Tracer, Nanofocus Inc., Republic of Korea) was used to characterize the electrical properties of the absorber surfaces. A Pt/Ir‐coated tip was used for KPFM and c‐AFM (Nanosensors, Inc., Switzerland). KPFM measurements were performed in noncontact mode with a set point at ≈30 nm, and c‐AFM measurements were performed in contact mode. TEM analysis was conducted on a JEM‐2100F (JEOL, Republic of Korea) high‐resolution transmission electron microscope coupled with EDX. The depth profiles of the CZTSSe solar cells were measured via SIMS (IMS‐7f, Cameca, USA).

##### Statistical Analysis

All values were expressed as mean ± standard error of the mean. Data were analyzed using analysis of variance (one‐way ANOVA) with post hoc Tukey test. A statistical software program (OriginPro 9.1, USA) was used and *p* < 0.05 was considered to be statistically significant.

## Conflict of Interest

The authors declare no Conflict of interest.

## Supporting information

Supporting Information
