# Supplementary material for: Impact of Na Doping on the Carrier Transport Path in Polycrystalline Flexible Cu2ZnSn(S,Se)4 Solar Cells
Source: Adv Sci (Weinh). 2020 Sep 27;7(21):1903085. doi: 10.1002/advs.201903085 (PMC7610331; doi:10.1002/advs.201903085)
Supplement: Supplementary file 1 — Supporting Information [file ADVS-7-1903085-s001.pdf]

WILEY-VCH

© 2020 Wiley-VCH GmbH

ADVANCED SCIENCE
Open Access

Supporting Information

for *Adv. Sci.,* DOI: 10.1002/advs.201903085

Impact of Na Doping on the Carrier Transport Path in Polycrystalline Flexible Cu2ZnSn(S,Se)4 Solar Cells

*Woo-Lim Jeong,1, Kyung-Pil Kim1, Juran Kim, Ha Kyung Park, Jung-Hong Min, Je-Sung Lee, Seung-Hyun Mun, Sung-Tae Kim, Jae-Hyung Jang, William Jo, and Dong-Seon Lee**

© 2020 Wiley-VCH GmbH

Supporting Information

Impact of Na doping on the carrier transport path in polycrystalline flexible $Cu_2ZnSn(S,Se)_4$ solar cells

*Woo-Lim Jeong[2], Kyung-Pil Kim[1], Juran Kim, Ha Kyung Park, Jung-Hong Min, Je-Sung Lee, Seung-Hyun Mun, Sung-Tae Kim, Jae-Hyung Jang, William Jo, and Dong-Seon Lee**

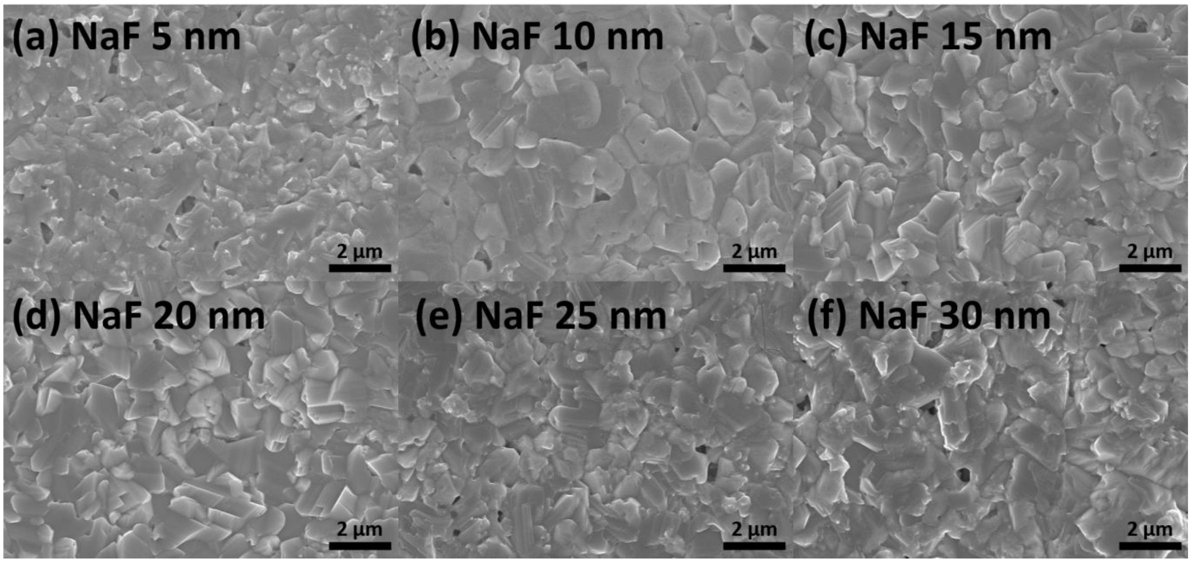

Figure S1. Surface scanning electron microscopy (SEM) images of the CZTSSe absorbing layers fabricated using different NaF layer thicknesses of a) 5 nm, b) 10 nm, c) 15 nm, d) 20 nm, e) 25 nm, and f) 30 nm.

Table S1. Elemental composition and compositional ratios of the CZTSSe absorbing layers.

	Elemental Composition					Compositional Ratio			
Sample	Cu (at%)	Zn (at%)	Sn (at%)	S (at%)	Se (at%)	Zn/Sn	Cu/ (Zn + Sn)	Se/S	(Se + S)/ Metal
CZTSSe NaF 5 nm	23	12.79	11.92	15.73	36.56	1.07	0.93	2.32	1.1
CZTSSe NaF 10 nm	21.71	12.22	11.62	17.65	36.8	1.05	0.91	2.08	1.2
CZTSSe NaF 15 nm	23.27	12.84	11.94	17.71	34.23	1.08	0.94	1.93	1.08
CZTSSe NaF 20 nm	21.96	12.83	11.8	16.46	36.95	1.09	0.89	2.24	1.15
CZTSSe NaF 25 nm	21.88	13.12	11.47	15.45	38.08	1.14	0.89	2.46	1.15
CZTSSe NaF 30 nm	21.48	13.71	10.85	17.45	36.51	1.26	0.87	2.09	1.17

[1] These authors contributed equally to this work.

[2] These authors contributed equally to this work.

Table S2. Solar cell parameters obtained from J-V curves and EQE data of the CZTSSe solar cells samples with varied NaF layer thicknesses.

Sample	PCE (%)	$J_{SC,\ J\text{-}V}$ (mA/cm^2)	V_{OC} (V)	R_S (Ωcm^2)	FF (%)	$J_{SC,\ EQE}$ (mA/cm^2)	E_g (eV)	$Eg/q-V_{OC}$ (V)
CZTSSe NaF 5 nm	0.69	13.97	0.176	10.1	27.88	15.93	1.18	1.004
CZTSSe NaF 10 nm	4.56	29.96	0.337	3.34	45.15	29.75	1.16	0.823
CZTSSe NaF 15 nm	7.41	33.71	0.417	3.18	52.67	33.67	1.14	0.723
CZTSSe NaF 20 nm	7.55	32.45	0.427	3.28	54.45	33.41	1.14	0.713
CZTSSe NaF 25 nm	7.75	31.8	0.427	3.0	57.03	32.04	1.15	0.722
CZTSSe NaF 30 nm	7.19	30.99	0.427	3.49	54.27	31.15	1.16	0.733
CZTSSe NaF 25 nm*	8.66	32.27	0.428	1.93	62.63			

*Data certified by the Korea Institute of Energy Research (KIER). The active area of each cell was 0.187 cm^2.

(별표 제4호 나-02)

Test Results

Report No. : KIER-19-0122

Page(4) / (4)Pages

[Appendix 2]

Gwangju Institute of Science and Technology (GIST)
CZTSSe solar cell

Device ID : CZTSSe #2
Date of Test : March 19, 2019
Simulator : WACOM, WXS-155S-L2 (Class-AAA)
Reference solar cell : KIER-SS-UF #1
Test condition : STC (AM1.5G, 100 mW/cm², (25.0 ± 1.0) °C)

Device Area : 0.187 29 cm² (active area)
Sample Type : CZTSSe solar cell (metal foil substrate)

한국에너지기술연구원
KOREA INSTITUTE OF ENERGY RESEARCH

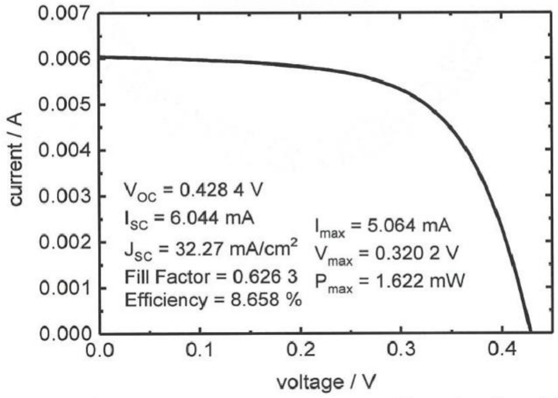

Operator: SangMin Lee

Photovoltaics Laboratory, Korea Institute of Energy Research
152, Gajeong-ro, Yuseong-gu, Daejeon, 34129, Korea
Tel : +82-42-860-3182, e-mail : notask@kier.re.kr

Figure S2. Certificated PCE of the best-performing CZTSSe solar cell (with a 25 nm-thick NaF layer) on flexible Mo foil.

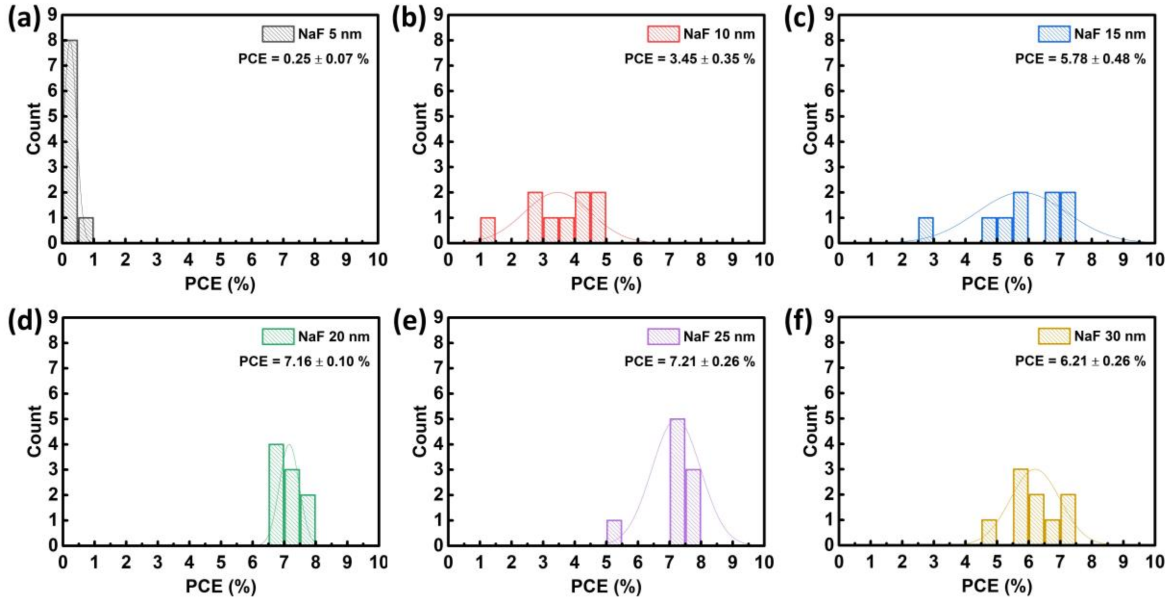

Figure S3. Histograms of PCE with fitted normal distribution curves of the CZTSSe solar cells with varied NaF layer thicknesses (n = 9). All statistical data were expressed as the mean ± standard error of the mean.

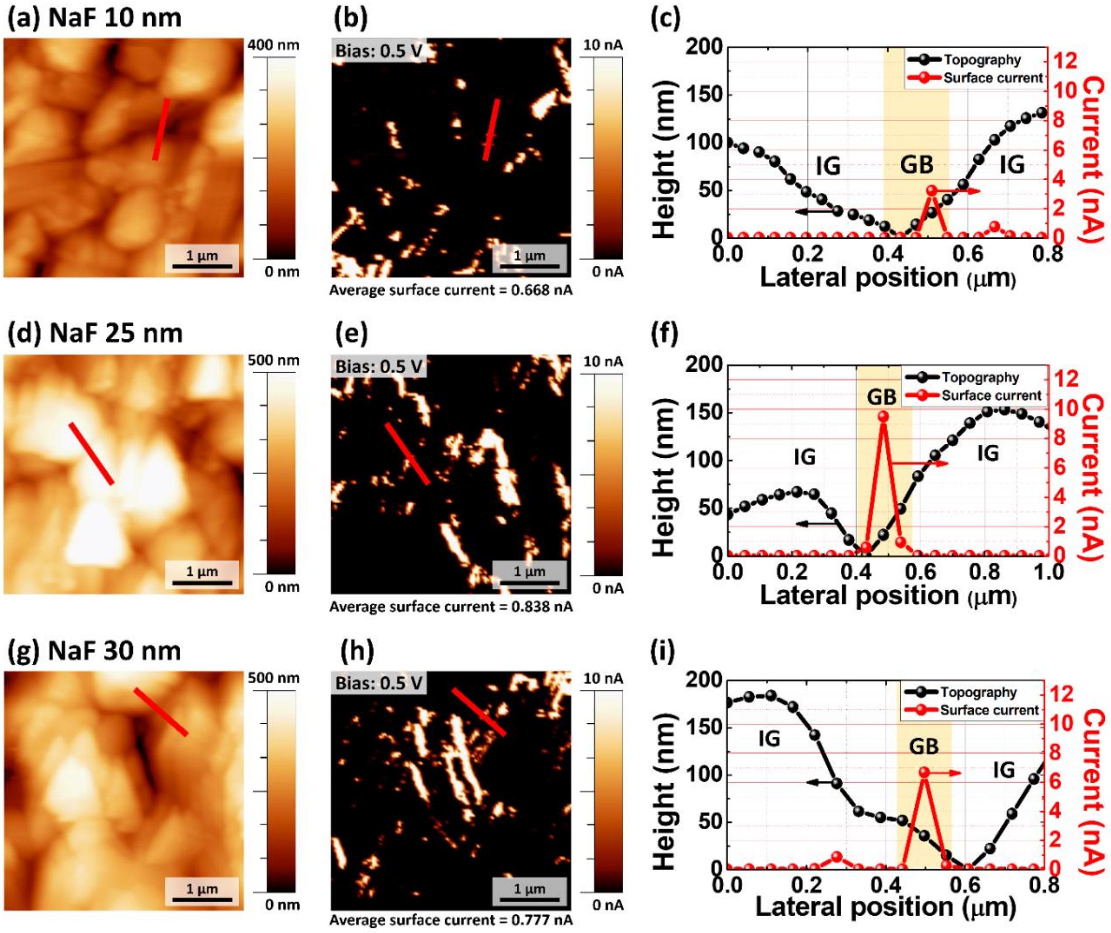

Figure S4. Surface topography, local current characteristics, and line profiles of the CZTSSe absorbing layers for samples with NaF layer thicknesses of a–c) 10 nm, d–f) 25 nm, and g–i) 30 nm determined by conductive atomic force microscopy (c-AFM) measurements.

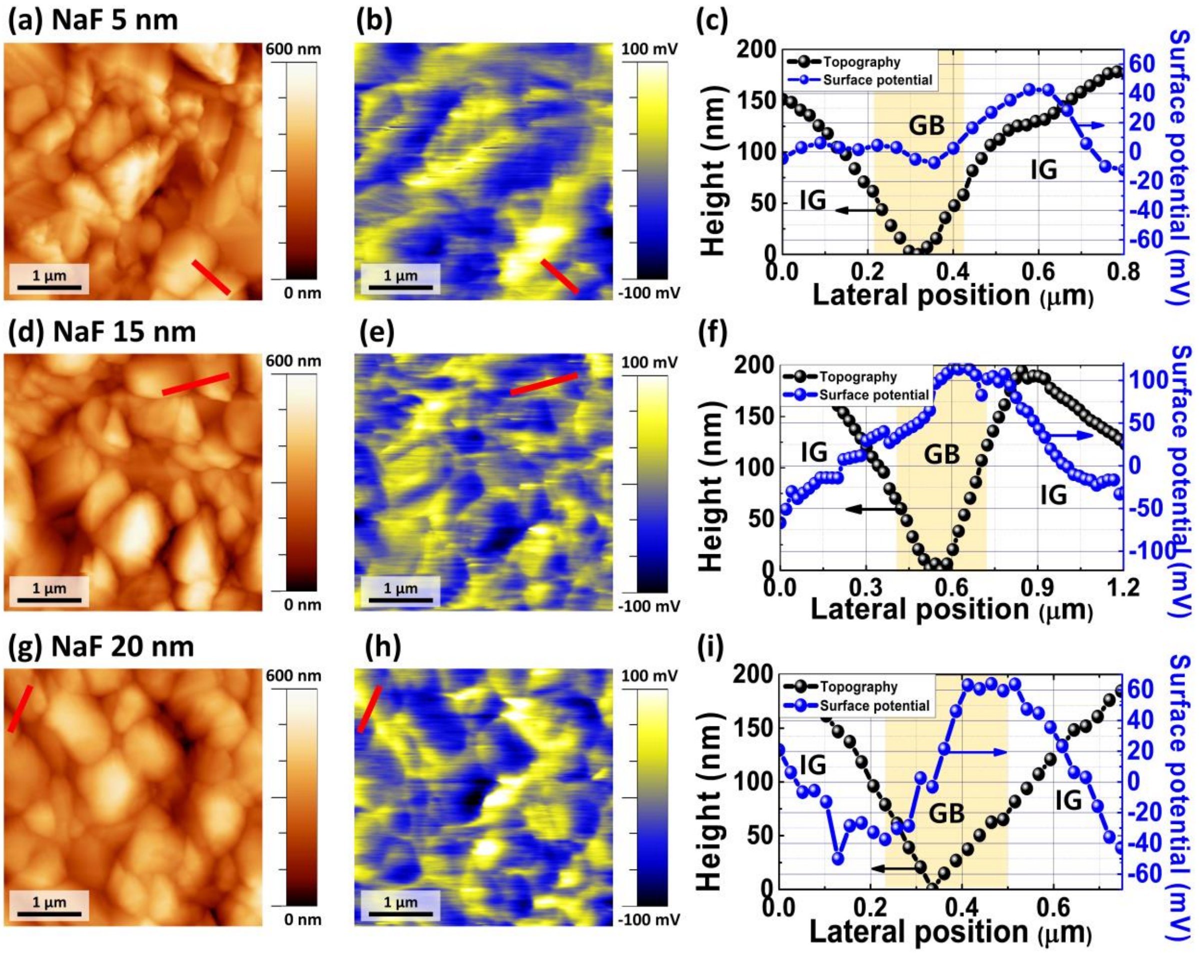

Figure S5. Surface topography, potential characteristics, and line profiles of the CZTSSe absorbing layers with a–c) 5 nm, d–f) 15 nm, or g–i) 20 nm NaF layer thicknesses determined from Kelvin probe force microscopy (KPFM) measurements.

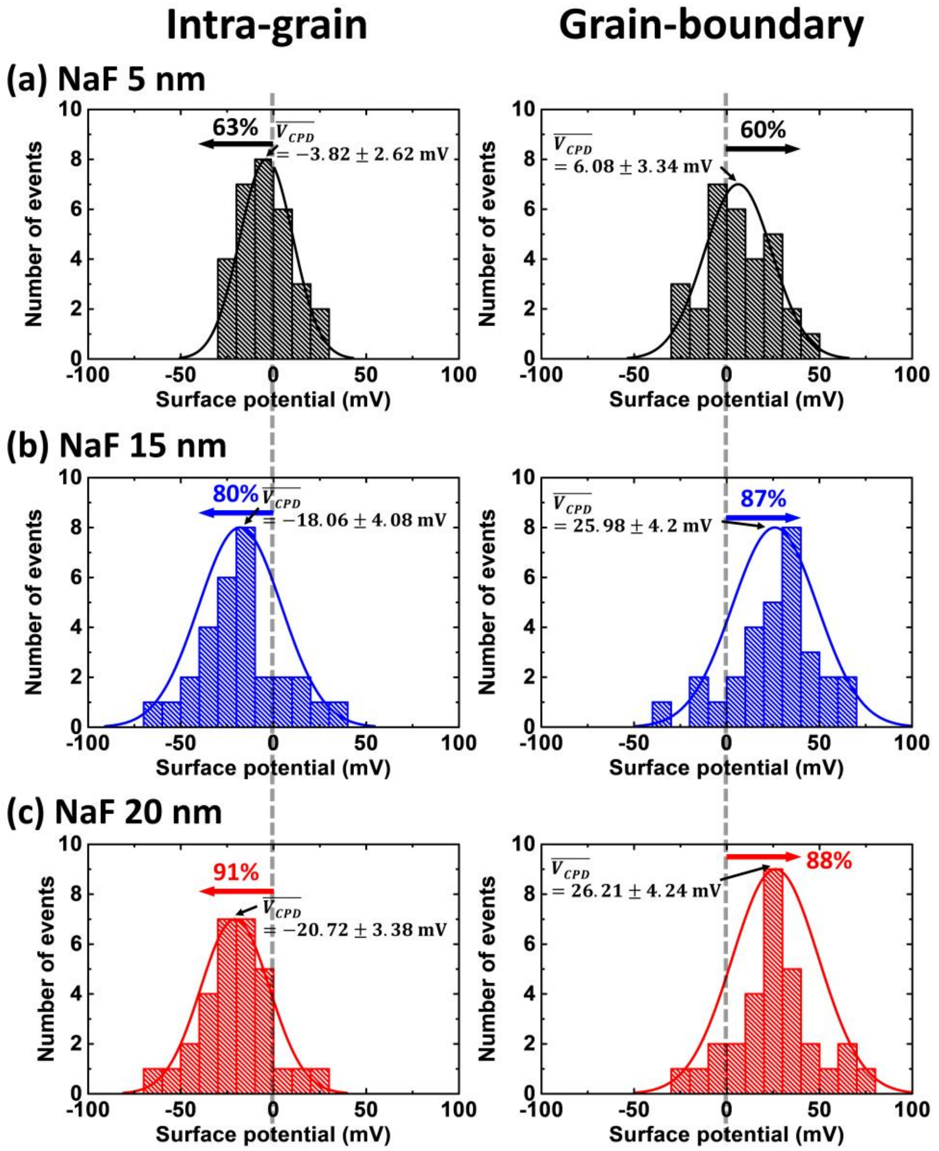

Figure S6. Histograms of the intra-grain (IG) and grain-boundary (GB) surface potential distributions obtained from the line profile data of the KPFM measurements (n = 30). Data are presented as mean ± standard error of the mean from three samples.

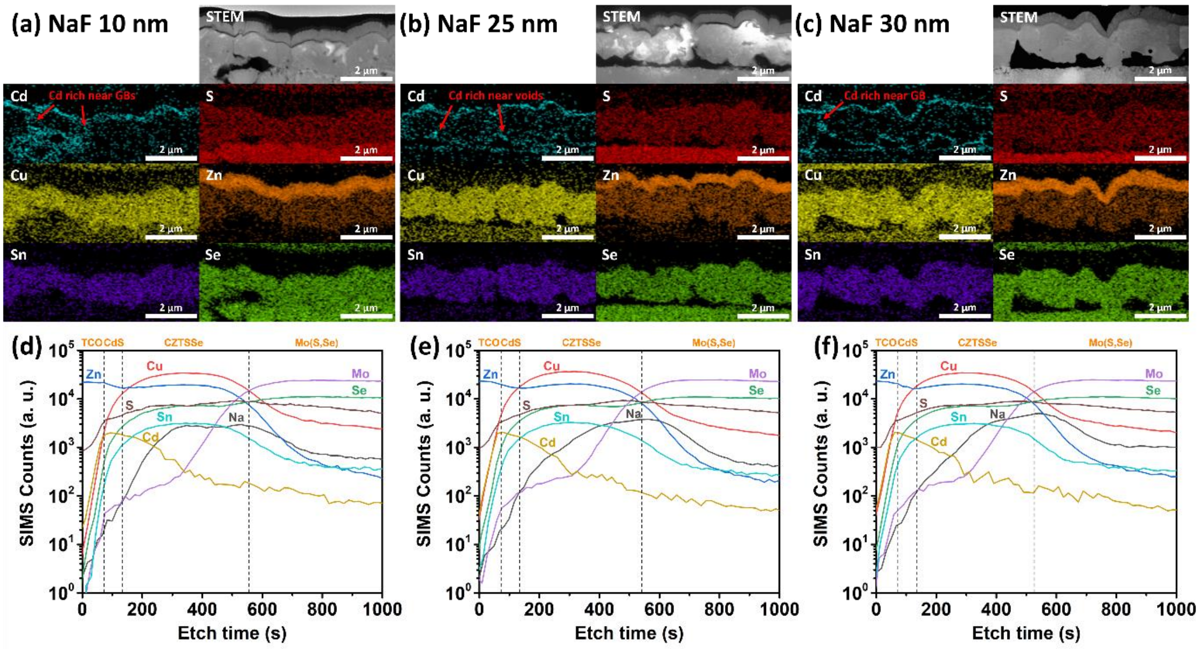

Figure S7. Transmission electron microscopy (TEM) images and the corresponding energy-dispersive x-ray spectroscopy (EDX) mapping images of the CZTSSe solar cell samples with NaF thicknesses of a) 10 nm, b) 25 nm, and c) 30 nm. Secondary ion mass spectrometry (SIMS) depth compositional profiles of the relevant elements in CZTSSe solar cell samples with NaF thicknesses of d) 10 nm, e) 25 nm, and f) 30 nm.

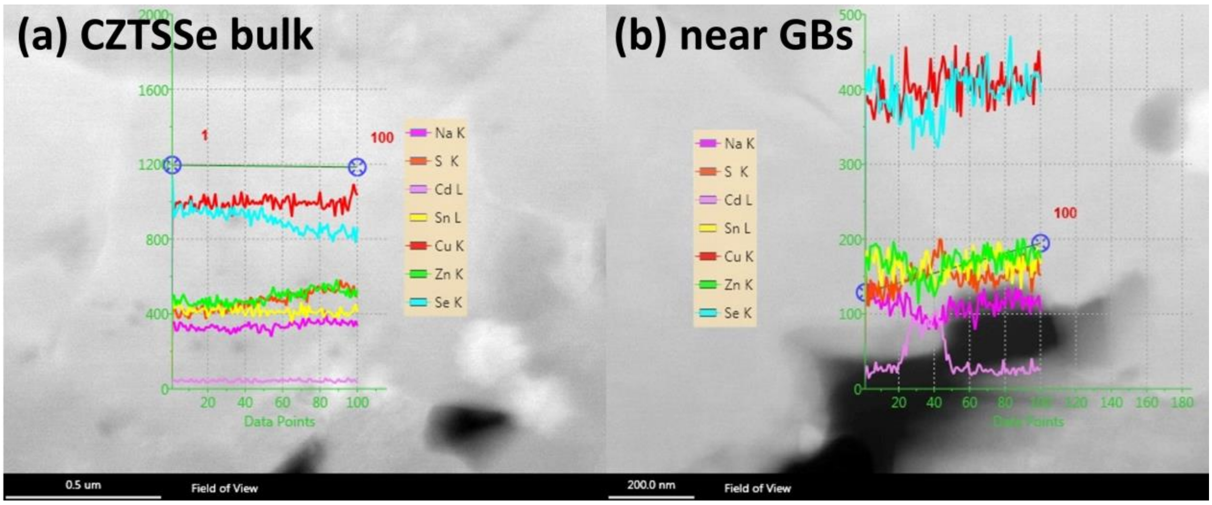

Figure S8. TEM image and the EDX line profile of the CZTSSe solar cell a) at the bulk and b) near the GBs of the CZTSSe solar cells with a NaF thickness of 25 nm. It appears that the Cd atoms replaced the Zn atoms near the GBs to form $Cu_2(Zn,Cd)Sn(S,Se)_4$.

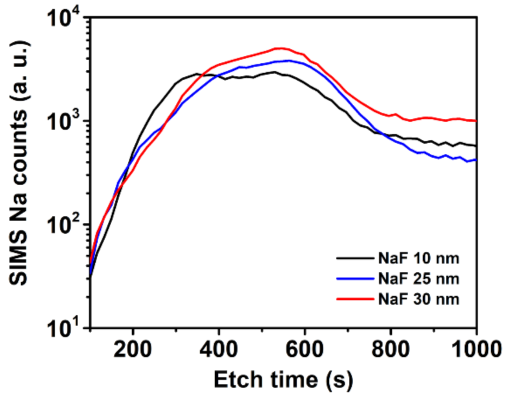

Figure S9. Secondary ion mass spectrometry (SIMS) depth compositional profiles of in CZTSSe solar cell samples with varying thicknesses of NaF.

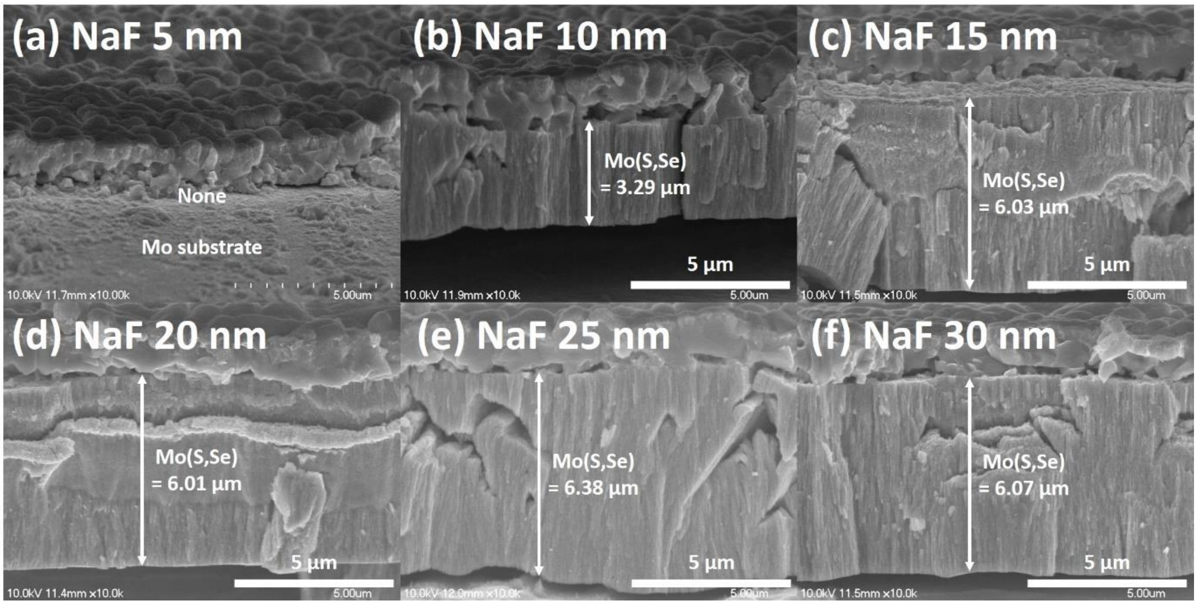

Figure S10. Cross-sectional SEM images of the CZTSSe solar cells fabricated using different NaF layer thicknesses of a) 5 nm, b) 10 nm, c) 15 nm, d) 20 nm, e) 25 nm, or f) 30 nm.
